# Spatial expression of IKK-alpha is associated with a differential mutational landscape and survival in primary colorectal cancer

**DOI:** 10.1038/s41416-022-01729-2

**Published:** 2022-02-16

**Authors:** Meera Patel, Kathryn A. F. Pennel, Jean A. Quinn, Hannah Hood, David K. Chang, Andrew V. Biankin, Selma Rebus, Antonia K. Roseweir, James H. Park, Paul G. Horgan, Donald C. McMillan, Joanne Edwards

**Affiliations:** 1 Wolfson Wohl Cancer Research Centre, Institute of Cancer Sciences, University of Glasgow, Glasgow, UK; 2 School of Medicine, Wolfson Medical School Building, University of Glasgow, Glasgow, UK; 3 Academic Unit of Surgery, School of Medicine, University of Glasgow, Royal Infirmary, Glasgow, UK

**Keywords:** Prognostic markers, Colorectal cancer

## Abstract

**Background:**

To understand the relationship between key non-canonical NF-κB kinase IKK-alpha(α), tumour mutational profile and survival in primary colorectal cancer.

**Methods:**

Immunohistochemical expression of IKKα was assessed in a cohort of 1030 patients who had undergone surgery for colorectal cancer using immunohistochemistry. Mutational tumour profile was examined using a customised gene panel. Immunofluorescence was used to identify the cellular location of punctate IKKα expression.

**Results:**

Two patterns of IKKα expression were observed; firstly, in the tumour cell cytoplasm and secondly as discrete ‘punctate’ areas in a juxtanuclear position. Although cytoplasmic expression of IKKα was not associated with survival, high ‘punctate’ IKKα expression was associated with significantly reduced cancer-specific survival on multivariate analysis. High punctate expression of IKKα was associated with mutations in KRAS and PDGFRA. Dual immunofluorescence suggested punctate IKKα expression was co-located with the Golgi apparatus.

**Conclusions:**

These results suggest the spatial expression of IKKα is a potential biomarker in colorectal cancer. This is associated with a differential mutational profile highlighting possible distinct signalling roles for IKKα in the context of colorectal cancer as well as potential implications for future treatment strategies using IKKα inhibitors.

## Introduction

Colorectal cancer (CRC) is the third most common cause of death worldwide. The heterogeneity observed in CRC is reflected in how patients present, how they respond to treatment and survival outcomes. In the United Kingdom, the 5-year survival is ~60% [1]. Although there has been a widespread expansion in the molecular genetics of CRC, it remains a challenge to translate advances in CRC genomics into clinically relevant prognostic or predictive tests. To expand this approach, investigation of molecular pathways and processes downstream of mutational events in patient tissue samples offers an avenue to identify subgroups of patients, identify prognostic or predictive biomarkers whilst simultaneously uncovering novel therapeutic targets.

One such pathway is nuclear factor kappa-light-chain enhancer of activated B cells (NF-κB) which plays essential physiological roles, including regulation of inflammation and immunity, lymphocyte differentiation, cell specialisation and maintenance of epithelial integrity, for example, in the gastrointestinal tract. There is a large body of evidence implicating NF-κB in all aspects of CRC tumorigenesis, from early adenoma to invasive cancer and metastasis. NF-κB has also been associated with treatment resistance in widely used therapies. It should be noted that the majority of this evidence originates from studies based on cell lines and animal models [2] with limited understanding of expression in human CRC tissue and how this may be related to patient outcomes. Moreover, these studies focus on the canonical arm of NF-κB signalling which results in recruitment of the IKK complex composed of subunits IκB kinase-alpha (IKKα), IκB kinase-beta (IKKβ) and IκB kinase-gamma (IKKγ)/NEMO and subsequent phosphorylation-induced degradation of IκB inhibitory protein which results in unmasking of the nuclear localising sequence (NLS) of the p50:p65 complex. Once the NLS is unmasked, this heterodimer can enter the nucleus where it regulates gene transcription. In contrast, activation of the non-canonical arm of NF-κB pathway arises as a result of NF-κB inducing kinase (NIK) stabilisation, which subsequently phosphorylates IKKα and results in phosphorylation-dependent ubiquitination of NF-κB precursor protein p100. This liberates the p52 subunit which then forms a heterodimer complex with RelB. The p52:RelB complex translocates to the nucleus where it regulates gene transcription. The role of the non-canonical pathway and specifically key regulatory kinase IKKα is not understood in CRC.

In other cancers, for example, oestrogen receptor-positive breast cancer, high expression of IKKα is associated with reduced time to recurrence and reduced cancer-specific survival [3], low expression is an independent predictive biomarker for lower recurrence on sequential therapy [4]. Activation of IKKα has also been implicated in prostate cancer tumorigenesis [5, 6]. There is evidence that implicates IKKα in CRC tumorigenesis independent of traditional NF-κB activity [7–9]. Further work using in vitro models of CRC has shown deficiency of IKKα results in a reduction in the expression of genes essential for maintaining intestinal stem cell function. In addition, deficiency of IKKα in APC mutated mice can reduce tumour initiation and proliferation but is not required for normal tissue homeostasis suggesting IKKα is essential for tumour initiation but is non-essential for maintaining normal homeostasis, thereby making IKKα a clinically exploitable target [10]. Toxicity associated with inhibitors of IKKβ has hampered the emergence of these compounds in the clinical setting. However, first-in-class selective inhibitors of IKKα have been reported and progress to pre-clinical patient testing is awaited [11, 12]. In this study we aim to investigate the relationship between expression of IKKα in patients undergoing surgery for CRC, mutational profile, tumour phenotype and features of the tumour microenvironment, and patient survival.

## Patients and methods

A cohort of 1030 patients with stage I–IV CRC who have undergone surgical resection for CRC within Glasgow hospitals was available for inclusion. 758 patients were identified retrospectively and 272 were identified from a prospectively maintained database of patients who had undergone surgery for CRC. All patients underwent surgery between 1997 and 2007. Patient cohort characteristics are outlined in Table 1. Patient exclusions were based on the following criteria: surgery with palliative intent, surgery for inflammatory bowel disease-related malignancy, neoadjuvant chemotherapy (excluded due to potential immunological impact on the tumour microenvironment), familial cancer syndrome, or mortality within 30 days of surgery. Patients with tumours proximal to the splenic flexure were considered right-sided. Tumours were staged according to the 5th edition of the AJCC TNM classification with additional data retrieved from pathological reports issued after resection. Following surgery patients were discussed at a local multi-disciplinary meeting. Patients undergoing colonic or rectal surgery with stage III or high-risk stage II disease without significant comorbidity were offered 5-fluorouracil-based adjuvant chemotherapy based on guidelines at the time.

**Table 1:** IKKα expression, associations with clinicopathological characteristics and the tumour microenvironment in patients undergoing surgery for colorectal cancer.

		All *n* = 695	Low cytoplasmic IKKα expression *n* = 550	High cytoplasmic IKKα expression *n* = 145	*p*
Host characteristics					
Age (*n* = 695)	<65	214 (31)	176 (32)	38 (25)	0.115
	>65	481 (69)	370 (68)	111 (75)	
Sex (*n* = 695)	Female	343 (49)	269 (49)	74 (50)	0.932
	Male	352 (51)	277 (51)	75 (50)	
Type of surgery (*n* = 694)	Elective	555 (80)	438 (80)	117 (79)	0.621
	Emergency	139 (20)	107 (20)	32 (21)	
Tumour location (*n* = 691)	Right	293 (42)	228 (42)	65 (44)	0.345
	Left	246 (36)	190 (35)	56 (38)	
	Rectum	152 (22)	126 (23)	26 (18)	
Tumour characteristics					
T stage (*n* = 695)	1–2	115 (17)	98 (18)	17 (12)	0.022
	3	382 (55)	301 (55)	81 (54)	
	4	198 (28)	147 (27)	51 (34)	
N stage (*n* = 693)	0	433 (63)	343 (63)	90 (60)	0.188
	1	184 (27)	148 (27)	36 (24)	
	2	76 (10)	53 (10)	23 (16)	
TNM stage (*n* = 695)	I	94 (14)	81 (15)	13 (9)	0.263
	II	333 (48)	260 (47)	73 (50)	
	III	253 (36)	196 (36)	57 (39)	
	IV	15 (2)	13 (2)	2 (2)	
Tumour differentiation (*n* = 695)	Mod/well	623 (90)	495 (91)	128 (86)	0.092
	Poor	72 (10)	51 (9)	21 (14)	
Venous invasion (*n* = 695)	No	461 (66)	355 (65)	106 (71)	0.157
	Yes	234 (34)	191 (35)	43 (29)	
Margin involvement (*n* = 695)	No	654 (94)	512 (94)	142 (95)	0.471
	Yes	41 (6)	34 (6)	7 (5)	
Necrosis (*n* = 683)	Absent	417 (61)	335 (62)	82 (57)	0.211
	Present	266 (39)	203 (38)	63 (43)	
Proliferation (*n* = 690)	Low	282 (41)	211 (39)	71 (48)	0.057
	High	408 (59)	330 (61)	78 (52)	
MMR status (*n* = 676)	Competent	578 (84)	458 (84)	120 (81)	0.348
	Deficient	113 (16)	85 (16)	28 (19)	
Tumour microenvironment					
Klintrup-Mäkinen grade (*n* = 670)	Weak	461 (67)	263 (67)	98 (67)	0.959
	Strong	224 (33)	176 (33)	48 (33)	
Tumour stroma percentage (*n* = 660)	Low	522 (77)	403 (76)	119 (82)	0.174
	High	153 (23)	126 (24)	27 (18)	
Adjuvant therapy (*n* = 261)	No	166 (63)	129 (63)	37 (64)	0.938
	Yes	96 (37)	75 (37)	21 (36)	

This study has been conducted in accordance with the REMARK (Reporting recommendations for tumour marker prognostic studies) Guidelines (McShane L et al., *JNCI*, 2005) (Supplementary Table 1).

### Assessment of IKKα expression

Once antibody validation and optimisation were completed, tissue microarrays were requested from NHS Research Scotland Greater Glasgow and Clyde Biorepository and were stored at 4 °C. Using immunohistochemistry, the previously constructed tissue microarray comprising four 0.6 mm cores per patient of formalin-fixed paraffin-embedded cancer tissue was used to assess the expression of IKKα. Tissues were dewaxed in xylene for 5 min (×2) and rehydrated through a series of graded alcohols. Antigen retrieval was performed using citrate buffer at pH6 under pressure for 5 min. Endogenous peroxidase activity was blocked using 3% hydrogen peroxide for 10 min. Five percent horse serum was applied for 30 min as a blocking solution. Tissue microarrays were incubated overnight at 4 °C with anti-IKKα rabbit polyclonal antibody (GWB-66250 GenWay Biotech, California, USA) at a concentration of 1:1000. After washing in TBS, Envision^TM^ (K5007, Dako, Denmark) was applied at room temperature before washing again in TBS. DAB substrate was applied to tissues for 5 min until colour developed before washing in running water for 10 min. Tissues were counterstained in haematoxylin for 1 min and blued with Scott’s tap water substitute before being dehydrated through a series of graded alcohols. Slides were mounted with coverslips applied using histological mounting medium (Omnimount, National Diagnostics, Atlanta, USA).

### IKKα visualisation and scoring method

**Fig. 1:**
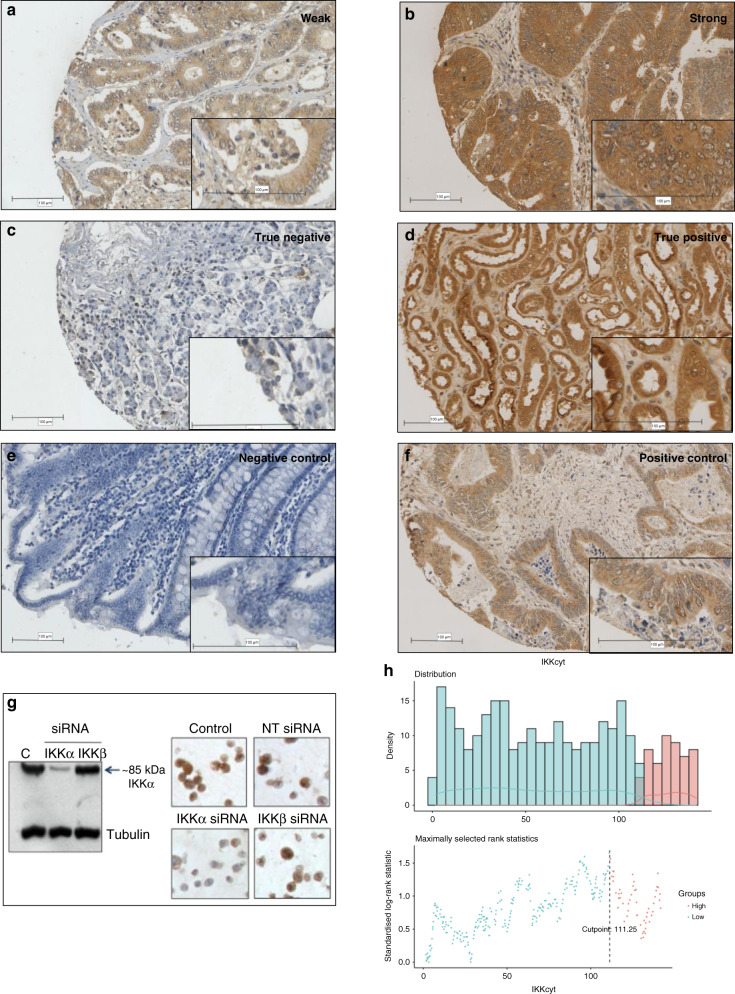
Representative images of IKKα immunohistochemical staining and controls. Example images of weak (**a**) and strong (**b**) cytoplasmic staining of IKKα. Representative images of true negative pancreatic tissue (**c**) and true positive kidney tissue (**d**) staining, respectively. Negative control colorectal tissue (**e**) with no antibody added during IHC staining process and colorectal tissue used as a positive control identified during optimisation (**f**). Western blot probed for IKKα and IKKβ using IKKα and IKKβ silenced lysates, MCF-7 cell pellets (IKKα silenced/IKKβ silenced) stained for IKKα as shown previously by Bennett et al. [3] (**g**). The optimal threshold for cytoplasmic IKKα histoscore was determined using R (**h**).

**Fig. 2:**
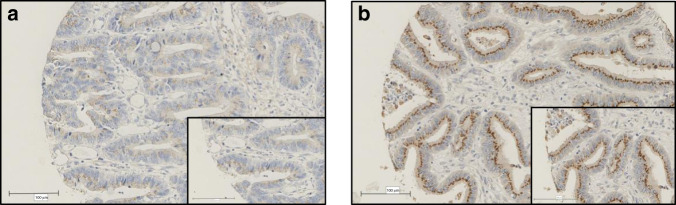
Representative images of punctate IKKα immunohistochemical staining and controls. Example images of low (**a**) and high (**b**) punctate staining of IKKα.

The Hamamatsu NanoZoomer (Welwyn Garden City, Hertfordshire, UK) was used to scan the sections at x20 magnification. The slides were then visualised using Slidepath Digital Image Hub, version 4.0.1 (Slidepath, Leica Biosystems, Milton Keynes, UK). Assessment of cytoplasmic IKKα expression was performed by a single-blinded examiner (MP) at ×20 magnification using the weighted histoscore. Histoscores were calculated from the sum of (1 × % cells staining weakly positive) + (2 × % cells staining moderately positive) + (3 × % cells staining strongly positive) with a maximum of 300. To assess for inter-observer variability, 10% of cores were co-scored by a second blinded examiner (HH) and the inter-class correlation coefficient (ICCC) was calculated in order to confirm the consistency between observers. Punctate expression of IKKα was graded as low or high depending on the number and size of puncta. There was a good correlation of scores between observers with ICCC scores of 0.77 for cytoplasmic and 0.70 for punctate expression of IKKα. (Examples of high and low cytoplasmic and punctate IKKα are shown in Figs. 1 and 2). Missing cores and those containing less than 10% tumour, were excluded from the analysis.

### Gene expressional profiling

Targeted capture sequencing was performed by Glasgow Precision Oncology Laboratory, University of Glasgow. RNA baits (Agilent) were utilised to capture a custom in-house designed panel of 151 cancer-associated genes (Supplementary Table 2). DNA was extracted from formalin-fixed paraffin-embedded sections from 237 stage I–IV CRC patients and standardised to a concentration of 4 ng/µl. Targeted capture libraries were prepared from 150 to 200 ng DNA. Sequencing was performed using an Illumina HiSeq 4000.

### Dual immunofluorescence and visualisation

Immunofluorescence was used to investigate a distinct pattern of IKKα expression. Dual immunofluorescence was used to investigate whether IKKα was localised to a specific cellular compartment. Tissues were dewaxed and rehydrated as described, however, dewaxing in xylene was increased to 10 min. A protease step prior to heat-induced antigen retrieval was performed using Protease Plus (322331, Advanced Cell Diagnostics, USA) for 30 min at 40 °C in a temperature-controlled humidifying chamber (HybEz II Oven^TM^, Advanced Cell Diagnostics, USA). Slides were cooled for 30 min before washing in running water for 10 min. 5% horse serum was applied for 30 min as a blocking solution. TMAs were incubated overnight at 4 °C with anti-IKKα rabbit polyclonal antibody (GWB-66250 GenWay Biotech, California, USA) at a concentration of 1:1000 and either anti-Golgi 58 mouse antibody (1:100, Abcam, ab27043), anti-Rab5 (1:100, Abcam ab66746) or anti-Rab7 (1:1000, Abcam, ab50533) before washing again in TBS. Tissues were incubated in 1:500 fluorescent secondary antibodies (AlexaFluor® 555 goat anti-rabbit, ThermoFisher, A2148 and Alexa Fluor® 488 goat anti-mouse, ThermoFisher, A11029) for 60 min at room temperature. Slides were rinsed once again in TBS and mounted using Vectashield Mounting Medium containing the nuclear counterstain DAPI (H-1200, Vector Laboratories, Burlingame, CA, USA). Wide-field epifluorescence images were acquired using ZEISS LSM 780 Confocal Microscope. Using a ×10 objective lens, images were taken with a ×40 oil immersion lens. ZEN 2 software (Zeiss, Germany) was used to visualise the images.

To ensure the pattern of staining observed with the Golgi 58 antibody was not the result of non-specific staining, an isotype control primary antibody (14-4714-82, ThermoFisher, UK) was used at the same concentration (1:100) as the primary antibody. There was no evidence of non-specific background staining with the isotype control. For the Golgi 58 marker, confocal images were acquired using a 40 × 1.3 numerical aperture Zeiss plan apochromat oil lens on a Zeiss LSM880 Airyscan microscope in Airyscan ‘super-resolution’ mode with sequential scanning through a different emission filter for the green and red channels to minimise cross talk. Airyscan processing was performed with default settings. Imaris (Bitplane) software was used to visualise the images.

### Statistical analysis

The optimal threshold for cytoplasmic IKKα histoscore was determined using R package ‘maxstat’. A weighted histoscore ≥111was considered ‘high’ expression (Fig. 1h). Punctate expression of IKKα could not be assessed using the weighted histoscore and was therefore graded as low or high expression depending on the number and size of these discrete areas. Pearson’s chi-squared test was used to assess associations between the expression of IKKα and clinicopathological characteristics. Kaplan–Meier log-rank survival analysis was used to examine associations with cancer-specific survival (CSS). Cox-proportional hazards regression was used to calculate hazard ratios (HR) and 95% confidence intervals (95% CI). Variables found to be statistically significant (*p* < 0.05) on univariate analysis were entered into a Cox regression multivariate model using a backward conditional method. A *p-*value of <0.05 was considered to be significant. Analyses were performed using SPSS software version 27 (IBM SPSS).

Gene mutation data were analysed and visualised using R package ‘maftools’. The 10 most commonly mutated genes are displayed and labelled in oncoplots. Fisher’s exact test was used to compare the 10 most statistically significant genes in patients with low and high expression of cytoplasmic and punctate IKKα, results are displayed using a co-bar plot and forest plot generated using the R package ‘maftools’. Lolliplop plots mapping mutations in the AR gene were generated using R package ‘ggplot2’. Code can be visualised in supplementary Table 3.

## Results

### Prognostic analysis of IKKα gene (CHUK) expression in TCGA dataset

Somatic mutation data related to the CHUK (IKKα) gene signature was extracted from The Cancer Genome Atlas (TCGA) panCancer atlas colorectal cohort. A total of 597 patients with information regarding cancer-specific survival were pooled together, Kaplan–Meier was used to estimate cancer-specific survival and univariate analyses were performed using the log-rank test. Statistical analyses were performed using cBioPortal. Patients with altered CHUK gene signatures had a statistically significant reduced cancer-specific survival outcome (*p* = 0.026) (Supplementary Fig. 1). Below describes the investigation of IKKα protein expression in 1030 patients who have undergone surgical resection of a primary CRC.

### Cohort characteristics

**Fig. 3:**
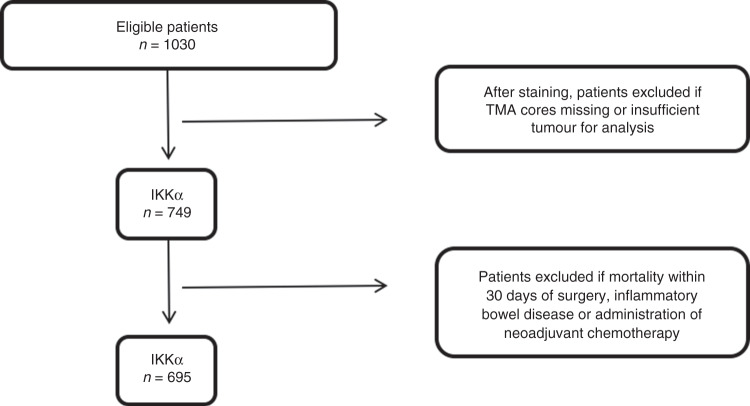
Flow diagram demonstrating patient exclusions. Patients with missing cores or insufficient tumour for analysis were excluded. Thereafter patients who had either died within 30 days of surgery, had inflammatory bowel disease-related malignancy or had received neoadjuvant chemotherapy were excluded from final analysis.

Following exclusions outlined in Fig. 3, 695 patients who had undergone surgical resection of a primary CRC were included. Baseline characteristics are summarised in Table 1. Median follow-up was 139 months (interquartile range 120–166 months) with 188 cancer-associated deaths and 233 non-cancer deaths. Median cancer-specific survival was 95 months (95% CI, 85.5–106.0).

In 237 patients, a total of 144 gene variants were identified with distribution across common drivers of CRC; mutated APC in 74%, TP53 in 59%, and KRAS in 45% of the total variants detected. These findings are in keeping with the evolutionary landscape of CRC. The median overall variant allele frequency (VAF) was 24%, ranging from 3% to 100%. The 237 samples yielded 2234 single-nucleotide variants in the ten most commonly mutated genes (Supplementary Fig. 2).

### Cytoplasmic IKKα expression in primary colorectal cancer

**Fig. 4:**
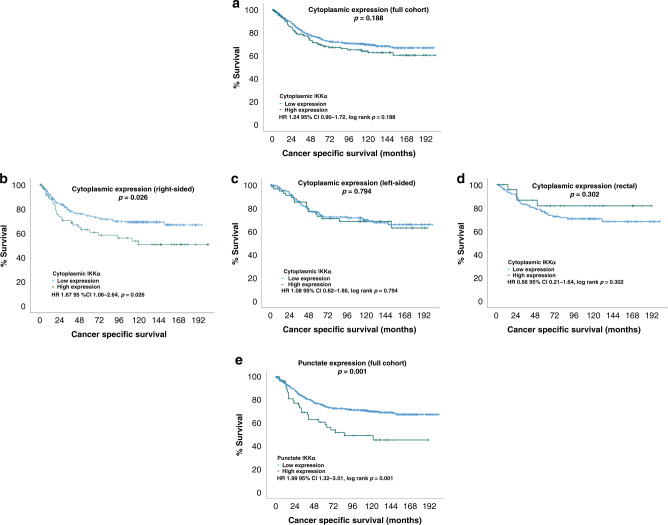
IKKα expression and survival. Expression of IKKα in the cytoplasm was not associated with CSS in the full cohort (**a**). High expression of cytoplasmic IKKα was associated with significantly worse cancer-specific survival in patients with right-sided tumours (**b**) but not in those with left-sided or rectal tumours (**c**, **d**). Punctate expression of IKKα was associated with CSS in the full cohort (**e**).

The protein expression of IKKα in tumour cell cytoplasm was assessed in 695 patients with a weighted histoscore range from 0 to 200. High protein expression of cytoplasmic IKKα was associated with increasing AJCC T stage (*p* = 0.022) but was not associated with patient age, sex, tumour location, nodal stage, tumour differentiation, venous invasion, margin involvement, tumour necrosis, proliferation index, tumour stroma percentage, inflammatory cell infiltrate (Klintrup-Mäkinen grade) or MMR status (Table 1). Protein expression of IKKα was not associated with cancer-specific survival (HR 1.24 95% CI 0.90–1.72, *p* = 0.188), in the full cohort, however, when examined in the context of tumour location, high expression of IKKα was associated with a significant reduction in cancer-specific survival in patients with right-sided tumour location (HR 1.67 95% CI 1.06–2.64, *p* = 0.026) (Fig. 4).

### Gene mutational profile in patients with low vs. high cytoplasmic IKKα expression

**Fig. 5:**
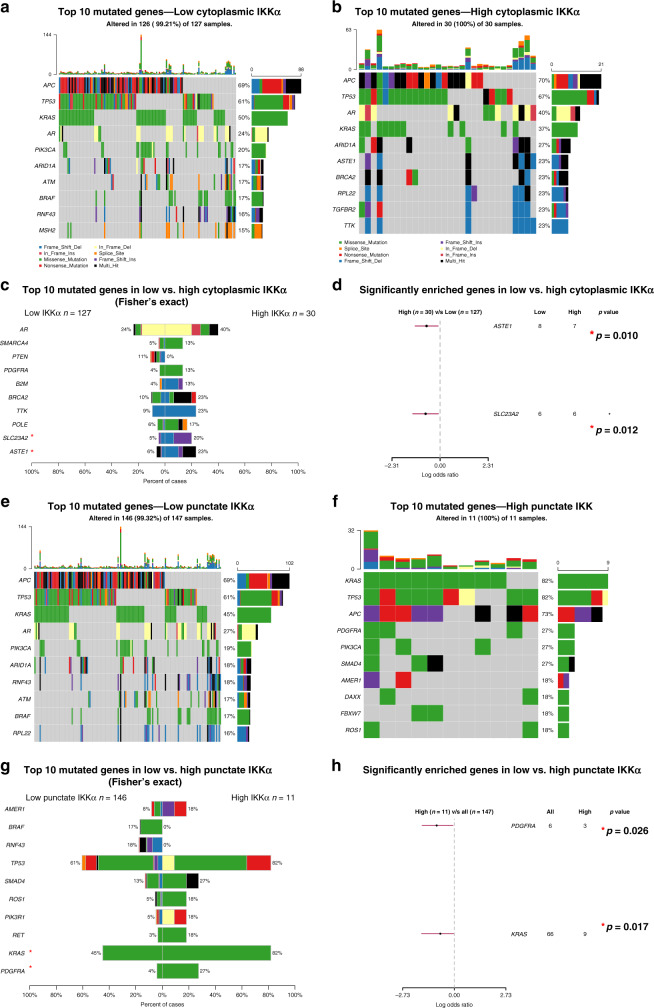
Mutational analysis. Oncoplots (**a**–**c**) and forest plots (**d**) demonstrating the top 10 mutated genes in low and high cytoplasmic IKKα. Oncoplots (**e**–**g**) and forest plots (**h**) demonstrating the top 10 mutated genes in low and high punctate IKKα.

The mutational profiles of patients with low and high protein expression of cytoplasmic IKKα were compared using 157 samples (Fig. 5a, b). There were 127 patients with low cytoplasmic IKKα expression and 30 patients with high expression. The top three mutations in patients with both low and high expression of IKKα were APC (69% vs. 70%), TP53 (61% vs. 67%) and KRAS (50% vs. 37%). Comparison of significantly mutated genes between the two groups using Fisher’s exact test (Fig. 5c), revealed that mutations in ASTE1 (6% vs. 23%, *p* = 0.010) and SLC23A2 (5% vs. 20%, *p* = 0.012) were significantly more enriched in patients with high cytoplasmic IKKα expression (Fig. 5d). Furthermore, a novel and potentially targetable mutation in AR was identified, this was enriched in patients with high IKKα expression (24% vs. 40%) although this did not reach statistical significance when the two groups were compared using Fisher’s exact test. The majority of AR mutations observed were due to in-frame deletions and within the N-terminal domain of AR (Supplementary Fig. 2C).

When oncogenic signalling pathways were compared, there was no difference in the enrichment of RTK-RAS, PI3K, cell cycle pathways, NOTCH, Wnt, TP53, TGF-β, NRF2, MYC or Hippo signalling pathways in patients with low vs. high cytoplasmic IKKα expression (Supplementary Fig. 3A).

### Punctate IKKα expression in primary colorectal cancer

An unexpected accumulation of IKKα in discrete juxtanuclear punctate areas was observed. Expression of punctate staining was variable; examples of low and high expression are shown in Fig. 2. Punctate expression was not associated with cytoplasmic IKKα expression (*p* = 0.506). Punctate expression was associated with younger age (<65) (*p* = 0.042) and male sex (*p* = 0.033) but not with tumour location, TNM stage, tumour differentiation, venous invasion, margin involvement, tumour necrosis, proliferation index, tumour stroma percentage, inflammatory cell infiltrate (Klintrup-Mäkinen grade) or MMR status. High punctate expression was observed in 56 (8%) patients and this was associated with a significant reduction in cancer-specific survival (HR 1.99 95% CI 0.90–1.72, *p* = 0.001) (Fig. 4). There was no association between cancer-specific survival when the expression of punctate IKKα was stratified by tumour location. On multivariate analysis; type of surgery (elective/emergency) (HR 1.69 95% CI 1.23–2.33, *p* = 0.002), TNM stage (HR 2.42 95% CI 1.90–3.08, *p* < 0.001), venous invasion (HR 1.51 95% CI 1.12–2.02, *p* = 0.007), margin involvement (HR 2.26 95% CI 1.43–3.57, *p* = 0.001), Klintrup-Mäkinen grade (HR 0.47 95% CI 0.32–0.68, *p* < 0.001), tumour stroma percentage (HR 1.58 95% CI 1.16–2.14, *p* = 0.005) and punctate IKKα (HR 1.97 95% CI 1.26–3.08, *p* = 0.006) were independently associated with reduced cancer-specific survival (Supplementary Table 4).

### Gene mutational profile in patients with low vs. high punctate IKKα expression

The mutational profiles of patients with low and high expression of punctate IKKα were compared using 157 samples (Fig. 5e, f). There were 146 patients with low punctate IKKα expression and 11 patients with high expression. The top three mutations in patients with both low and high punctate expression of IKKα were APC (69% vs. 73%), TP53 (61% vs. 82%) and KRAS (45% vs. 82%). Comparison of significantly mutated genes between the two groups using Fisher’s exact test (Fig. 5g) revealed that mutations in PDGFRA (4% vs. 27%, *p* = 0.026) and KRAS (45% vs. 82%, *p* = 0.017) were significantly enriched in patients with high punctate IKKα expression (Fig. 5h). Furthermore, 27% of patients with low punctate expression had mutations in the AR gene however no patients with high expression of punctate IKKα had AR mutations.

When oncogenic signalling pathways were compared, there was no difference in the enrichment of RTK-RAS, PI3K, cell cycle pathways, NOTCH, Wnt, TP53, TGF-β or NRF2, signalling pathways in patients with low vs. high punctate IKKα expression (Supplementary Fig. 3B). Of interest, all patients (100%) with high punctate IKKα expression observed at least one mutation in the RTK-KRAS pathway.

Other members of the non-canonical NF-κB pathway were also investigated. When the expression of NIK and RelB was examined, no association between the expression of these proteins with clinicopathological factors or survival was observed (results not shown).

### Investigation of punctate IKKα in the perinuclear space

Expression of punctate IKKα in the perinuclear space was associated with significantly reduced cancer-specific survival and a differential mutational profile in patients with low vs. high expression. Therefore, it was of interest to investigate the nature of this expression pattern further. A fluorescent pattern comparable to that observed with immunohistochemistry was identified in a number of patients. As with immunohistochemistry, there was a variation in the number and size of these discrete punctate areas. We hypothesised that the accumulation of IKKα was representative of IKKα localised within a specific cellular compartment. Markers of cellular compartments were investigated using dual fluorescence with IKKα.

**Fig. 6:**
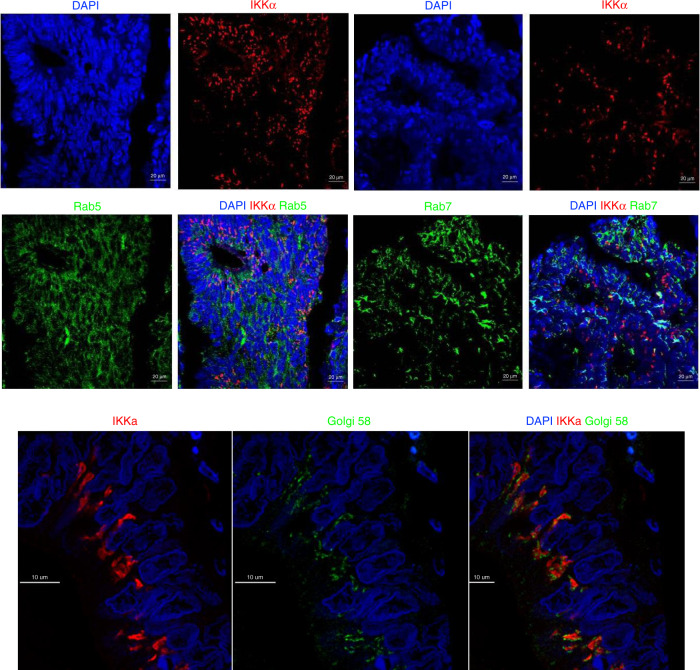
IKKα dual fluorescence with Rab5, Rab7 and Golgi 58. Co-localisation between IKKα (red) and Rab5 and Rab7 (green) was not observed in colorectal cancer tissue. DAPI (blue) represents nuclear counterstain. IKKα co-located with a Golgi marker (green) in colorectal cancer tissue.

Markers of the endosomal compartment (Rab5 and Rab7) and the Golgi apparatus were investigated (Fig. 6). There was no evidence of co-localisation between endosomal compartments and punctate IKKα. However, a well-established Golgi marker, Golgi 58 K, was observed to co-locate with punctate IKKα, where the Golgi marker appeared to closely surround IKKα punctate expression (Fig. 6). This observation raises a number of possible hypotheses and requires further investigation.

## Discussion

In this communication we report on the association between protein expression of key non-canonical NF-κB kinase, IKKα, patient and tumour characteristics and the mutational landscape in patients who have undergone surgery for CRC. We observed the cellular location of IKKα expression was associated with a differential mutational profile and survival outcome. Expression of IKKα was observed in two distinct patterns; firstly, in the tumour cell cytoplasm, which was expected, and secondly, in unexpected discrete juxtanuclear punctate areas. In the present study, cytoplasmic IKKα expression was not associated with survival; however, punctate IKKα expression was associated with a significant reduction in cancer-specific survival in patients who have undergone surgery for primary CRC. When the mutational profile of patients with low and high cytoplasmic IKKα expression was compared, we observed those with high expression of cytoplasmic IKKα had significant enrichment of ASTE1 and SLC23A2 when compared to those patients with low cytoplasmic IKKα expression. The role of these genes in CRC is not understood. The function of the ASTE1 gene is largely unknown however, it has a possible role in EGFR signalling [13]. Tougeron et al. report ASTE1 is one of the most frequently mutated genes in microsatellite unstable CRC and is associated with the density of tumour-infiltrating lymphocytes [14]. SLC23A2 encodes the sodium-dependent vitamin C transporter 2 (SVCT2). The SVCT2 transporter is found across most tissues including the colon [15] and its role in CRC is largely unknown. These observations require further investigation in CRC.

It was of interest that 40% of patients with high expression of cytoplasmic IKKα had a mutation within the AR gene compared to 24% of patients with low cytoplasmic IKKα expression although, this difference did not reach statistical significance. This may be attributed to only 30 patients expressing high cytoplasmic IKKα compared to 127 with low expression. When the mutational profile of patients with low and high punctate IKKα was compared, PDGFRA and KRAS were significantly enriched in patients with high punctate IKKα expression. To explore the mutational landscape, mutational analysis was only performed in a subgroup of 157 patients, 82% of patients with high punctate expression of IKKα had KRAS mutations and although this finding will require validation by future studies, the findings are clinically relevant particularly in light of limited treatments options for patients with KRAS mutations. Future studies should aim to validate this finding and examine the expression of IKKα in patients with and without KRAS mutations. A number of IKK inhibitors have been reported, although, none have made it into the clinical setting due to their broad-spectrum and non-specific nature. However, first-in-class IKKα specific inhibitors have been reported and will now be tested in mice [11, 12]. The present study supports the notion of targeting IKKα as a therapeutic target. Pre-clinical studies should aim to understand their efficacy particularly in the context of KRAS mutations.

We also observed 27% of patients with low punctate expression had mutations in the AR gene however, no patients with high expression of punctate IKKα had AR mutations. This did not reach statistical significance and may be attributed to only 10 patients with high IKKα punctate expression out of those who underwent mutational profiling. Nevertheless, this differential mutational profile suggests that IKKα signalling may be occurring through two independent mechanisms, one which is associated with AR mutation and the other which is associated with alterations in KRAS and inferior survival outcomes in primary colorectal cancer. A link between KRAS mutations and IKKα has been observed in lung adenocarcinoma development [16, 17]. For example, Song and co-workers observed lung-specific IKKα ablation in mince enhances KRAS-initiated lung adenocarcinoma development through a mechanism that regulates tumour cell-associated ROS metabolism. CHUK encodes IKKα; the same group observed patients with CHUK mutations had a median survival of 19.5 months compared to 44.6 months in the full cohort and this was potentiated in patients also carrying a KRAS mutation [17]. Therefore, to further investigate the relationship between KRAS and punctate IKKα expression observed in the present study, future studies in colorectal cancer should investigate the relationship between CHUK mutations, KRAS mutations and IKKα expression in patient samples.

The AR gene codes for the androgen receptor, a steroid hormone receptor that functions as a ligand-activated transcription factor and is activated by binding of androgenic hormones testosterone or dihydrotestosterone [18]. The androgen receptor has four domains: the amino-terminal activation domain (NTD), the DNA-binding domain (DBD), the hinge region (HR) and the carboxyl ligand-binding domain. The NTD comprises a significant part of the AR and is the least conserved of the four domains, facilitating essential AR functions required for AR activation. AR mutations have been widely studied in the context of prostate cancer and are associated with resistance to anti-androgen therapy [19]. AR truncations within the ligand-binding domain have been intensely investigated and are well documented to result in constitutively activated AR [20, 21]. However, in the present study mutations in AR were exclusively located within the NTD, although mutations in this region are not as well characterised, it has been reported that there is a transcription activation function within the NTD and a mutation within this region can result in transactivation of the AR. The AR has the ability to regulate the expression of different genes by employing its NTD as transcription activation units [22]. Although mutations within the NTD are rare in prostate cancer they are frequently reported in another disease such as Kennedys disease [22]. Therefore, although not a common mutation found in prostate cancer, its role in CRC could be highly significant especially as it is recognised that β-catenin has the ability to modulate the effects of AR by binding to the NTD, mutations in this region could therefore interfere with this process [23].

The role of androgens and AR mutations in the development of CRC is unclear although the presence of AR has been demonstrated in normal colonic mucosa and colorectal cancer [24–27]. A link between androgens and CRC risk has been previously reported. Data from clinical and non-clinical studies indicate androgens exert protective effects against the development of CRC. For example, Gillessen and co-workers reported long term androgen deprivation therapy for prostate cancer in over 100,000 men is associated with an increased risk of CRC. They observed a 30–40% increase in the rate of CRC among men with prostate cancer treated with androgen deprivation therapy compared to men with prostate cancer who were not. There was a dose-response effect with a higher risk of CRC with increasing duration of androgen deprivation, suggesting the association between androgen deprivation and CRC in this setting may be causal [28]. Animal studies have suggested androgen ablation promotes colon carcinogenesis and the administration of androgens is protective [29, 30]. The AR gene contains polymorphic trinucleotide CAG repeats demonstrated to inversely affect receptor transcriptional activity [31, 32]. Multiple somatic mutations of AR CAG repeats in colorectal carcinoma and adjacent normal mucosa, independent of MSI status, have been reported [33]. In a case-control study, the absence of AR expression and increasing length CAG repeats was associated with reduced 5-year overall survival in patients with CRC [34]. Although, in a separate case-control study, Rudolph and co-workers reported no association between AR CAG repeat polymorphisms and risk of CRC or any association between AR CAG repeat polymorphisms and survival after CRC diagnosis [35]. In the present study, AR mutations were observed in patients with low punctate IKKα expression but not in patients with high punctate IKKα expression. Therefore, we hypothesise that the presence of mutations in the AR gene is not only related to different IKKα signalling mechanisms but also the absence of such mutations confer a survival disadvantage by promoting tumorigenic activities of IKKα.

In the present study, punctate expression of IKKα was associated with a reduction in cancer-specific survival. Therefore, to further characterise the distribution of IKKα, markers of cellular transport were investigated using dual immunofluorescence. We hypothesised that the punctate expression of IKKα was representative of IKKα localised within a specific cellular compartment. Margalef and colleagues reported full-length IKKα undergoes cathepsin-dependent processing to produce a truncated isoform (p45-IKKα). They observed cathepsin co-localised with IKKα in ring-shaped structures corresponding to early endosomal marker Rab5. They also observed co-localisation between IKKα and late endosomal marker Rab7 but did not detect co-localisation between IKKα and autophagosomal marker LC3 or lysosomal marker LAMP1 [8]. Therefore, in the present study, we investigated markers of the endosomal compartment (Rab5 and Rab7) to understand if IKKα punctate staining corresponded to the staining pattern observed by Margalef and colleagues (Fig. 6).

Bowen and colleagues also observed a juxtanuclear dot-like pattern of staining in colorectal cancer tissue when investigating collagen expression with immunohistochemistry. They observed that this pattern of staining mirrored a staining pattern observed with a 58 K Golgi marker [36]. Therefore, in the present study, co-localisation of IKKα with a 58 K Golgi was investigated to understand if IKKα punctate staining corresponded to the staining pattern observed by Bowen and colleagues. Using an antibody raised against a resident Golgi enzyme (58 K, formiminotransferase cyclodeaminase), results of the present study showed IKKα was co-located with the Golgi apparatus or indeed a Golgi-related structure, where the Golgi marker appeared to closely surround IKKα punctate expression.

It is possible that co-location of IKKα with the Golgi marker represents an NF-κB independent role of IKKα. As discussed, Margalef and co-workers reported full-length IKKα undergoes cathepsin-dependent processing to produce a truncated isoform (p45-IKKα) in cytoplasmic vesicles, and that this truncated isoform of IKKα is implicated in CRC tumorigenesis in vivo [8, 37].

Alternatively, co-location of IKKα may represent a dysfunction of Golgi-related processes. For example, glycosylation is a key post-translational modification that takes place in the endoplasmic reticulum/Golgi network. Abnormal glycosylation has been associated with many human cancers. In CRC, Kellokumpu and co-workers reported a change in Golgi structure. They observed that in normal colorectal tissue the Golgi has a ‘horseshoe’-shaped configuration however, in cancer cells, this organisation is absent, and the Golgi appear as small punctate structures around, or close to the nuclei [38]. To date, phosphorylation of NF-κB subunits has been the most investigated post-translational modification, wider investigation of processes such as glycosylation may be required, particularly in CRC.

Although the Golgi complex is known as a central player in cellular transport, it is also an important site of key autophagy regulators [39]. Autophagy is the process whereby cellular components such as organelles or proteins are degraded and removed. Autophagy involves the formation of a double-membraned autophagasome containing the ‘cargo’ which undergoes fusion with the lysosome resulting in degradation of its contents. During an investigation into microglial activation after brain ischaemia, Li and co-workers observed SUMOylated ANXA1 overexpression promoted autophagosome formation, autolysosome maturation and specific autophagic degradation of IKKα. They described the co-localisation of IKKα with autophagy marker LC3 in discrete punctate areas. Specifically, they observed SUMOylated ANXA1 promoted the interaction of IKKα with the autophagy receptor NBR1 and this facilitated the selective autophagic degradation of IKKα [40]. Therefore, the co-location of punctate IKKα with the Golgi marker observed in the present study may represent the process of autophagic degradation of IKKα in response to dysregulated IKKα production. This requires further investigation but represents an area that could be exploited with further development of IKKα selective inhibitors.

In summary, in patients who have undergone surgery for colorectal cancer, the spatial expression of IKKα is associated with differential survival outcomes and mutational tumour profile. Results of the present study suggest different mechanisms of IKKα signalling in colorectal cancer and this requires further investigation. The predictive capacity of IKKα was not examined in the present study and future studies should aim to assess chemotherapeutic efficacy based on IKKα expression. Interestingly, a distinct pattern of punctate IKKα expression was observed and the nature of this requires further investigation.

## Supplementary information

Supplementary Table 1

Supplementary Tables and Figures

Reproducibility checklist
