# Supplementary material for: Spatial expression of IKK-alpha is associated with a differential mutational landscape and survival in primary colorectal cancer
Source: Br J Cancer. 2022 Feb 16;126(12):1704–14. doi: 10.1038/s41416-022-01729-2 (PMC9174220; doi:10.1038/s41416-022-01729-2)

Supplementary Tables and Figures

Supplementary Table 2. 151 cancer-associated gene panel

ABCB1	CSF1R	FGFR4	KMT2A	PHF6	SLC22A1	ZMYM3
ABCC2	CTNNB1	FLT1	KRAS	PIK3CA	SLC22A2	
ABL1	CYP19A1	FLT3	LAMA2	PIK3R1	SLC31A1	
ABL2	CYP2A6	FLT4	LCK	PSMB1	SLC34A2	
AKT1	CYP2B6	FSTL5	LTK	PSMB2	SLC45A3	
AKT2	CYP2C19	GNA11	MAP2K1	PSMB5	SLCO1B1	
AKT3	CYP2C9	GNAQ	MAP2K2	PSMD1	SMAD4	
ALK	CYP2D6	GNAS	MAP2K4	PSMD2	SMARCA4	
APC	DDR1	GSTP1	MAP3K1	PTCH1	SMARCB1	
ASXL1	DDR2	H3F3A	MAPK1	PTEN	SMO	
ATM	DDX3X	HNF1A	MED13	PTPN11	SNCAIP	
ATRX	DNMT3A	HRAS	MET	RAF1	SOS1	
BRAF	DPYD	IDH1	MLH1	RARA	SPRED1	
BRCA1	EGFR	IDH2	MPL	RARB	SRC	
BRCA2	ERBB2	IKZF1	MST1R	RARG	STK11	
CBL	ERBB3	IL2RA	MTOR	RB1	SUFU	
CDA	ERBB4	IL2RB	MYC	RET	TAS2R38	
CDH1	ERG	IL2RG	MYD88	ROS1	TET2	
CDKN2A	ESR1	INPP4B	NELL2	RPS6KB1	TP53	
CDKN2B	ESR2	JAK1	NF1	RUNX1	TRRAP	
CEBPA	EZH2	JAK2	NOTCH1	RXRA	TYK2	
CHD7	FBXW7	JAK3	NPM1	RXRB	UGT1A1	
CHIC2	FGFR1	KDM6A	NRAS	RXRG	VHL	
CREBBP	FGFR2	KDR	PDGFRA	SHH	WT1	
CRLF2	FGFR3	KIT	PDGFRB	SHOC2	YES1	

Supplementary Table 3. Supplementary Code

```
R version 4.0.2 (2020-06-22) -- "Taking Off Again"
Copyright (C) 2020 The R Foundation for Statistical Computing
Platform: x86_64-apple-darwin17.0 (64-bit)

R is free software and comes with ABSOLUTELY NO WARRANTY.
You are welcome to redistribute it under certain conditions.
Type 'license()' or 'licence()' for distribution details.

  Natural language support but running in an English locale

R is a collaborative project with many contributors.
Type 'contributors()' for more information and
'citation()' on how to cite R or R packages in publications.

Type 'demo()' for some demos, 'help()' for on-line help, or
'help.start()' for an HTML browser interface to help.
Type 'q()' to quit R.

[R.app GUI 1.72 (7847) x86_64-apple-darwin17.0]

[Workspace restored from /Users/meerapatel/.RData]
[History restored from /Users/meerapatel/.Rapp.history]

> install.packages("maxstat")
--- Please select a CRAN mirror for use in this session ---
trying URL 'https://cran.ma.imperial.ac.uk/bin/macosx/contrib/4.0/maxstat_0.7-25.tgz'
Content type 'application/x-gzip' length 180958 bytes (176 KB)
==================================================
downloaded 176 KB


The downloaded binary packages are in
	/var/folders/s4/vgt0mphs693dd3ztkl4kcfh80000gn/T//Rtmp7z01jt/downloaded_packages
> install.packages("survival")
trying URL 'https://cran.ma.imperial.ac.uk/bin/macosx/contrib/4.0/survival_3.2-11.tgz'
Content type 'application/x-gzip' length 6803373 bytes (6.5 MB)
==================================================
downloaded 6.5 MB


The downloaded binary packages are in
	/var/folders/s4/vgt0mphs693dd3ztkl4kcfh80000gn/T//Rtmp7z01jt/downloaded_packages
> install.packages("survminer")
trying URL 'https://cran.ma.imperial.ac.uk/bin/macosx/contrib/4.0/survminer_0.4.9.tgz'
Content type 'application/x-gzip' length 3227677 bytes (3.1 MB)
==================================================
downloaded 3.1 MB


The downloaded binary packages are in
	/var/folders/s4/vgt0mphs693dd3ztkl4kcfh80000gn/T//Rtmp7z01jt/downloaded_packages
> install.packages("tidyverse")
trying URL 'https://cran.ma.imperial.ac.uk/bin/macosx/contrib/4.0/tidyverse_1.3.1.tgz'
Content type 'application/x-gzip' length 421035 bytes (411 KB)
==================================================
downloaded 411 KB
```

```
The downloaded binary packages are in
                    /var/folders/s4/vgt0mphs693dd3ztkl4kcfh80000gn/T//Rtmp7z01jt/downloaded_packages
> library(maxstat)
> library(survival)
> library(survminer)
Loading required package: ggplot2
Loading required package: ggpubr
> library(tidyverse)
── Attaching packages ──────────────────────────────────────────────────────────
───────────────────────────────────────────────────────────── tidyverse 1.3.1 ──
✓ tibble  3.1.2     ✓ dplyr   1.0.7
✓ tidyr   1.1.3     ✓ stringr 1.4.0
✓ readr   1.4.0     ✓ forcats 0.5.1
✓ purrr   0.3.4
── Conflicts ───────────────────────────────────────────────────────────────────
──────────────────────────────────────────────────────────── tidyverse_conflicts() ──
✖ dplyr::filter() masks stats::filter()
✖ dplyr::lag()    masks stats::lag()
> mydata<-read.csv(file.choose())
> res.cut <- surv_cutpoint(mydata, time= "CSS", event="acd", variables = c("cyt"))
> summary(res.cut)
    cutpoint statistic
cyt   111.25  1.350276
>
> plot(res.cut,"cyt")

> library(maftools)
> IKKlow <- read.maf(file.choose())
-Reading
-Validating
--Removed 115 duplicated variants
--Non MAF specific values in Variant_Classification column:
  Multi_Hit
  3UTR
  inframe_indel|stop_gained
  inframe_indel
  inframe_indel|Missense_Mutation
--Non MAF specific values in Variant_Type column:
  Complex
-Silent variants: 338
-Summarizing
-Processing clinical data
--Missing clinical data
-Finished in 13.5s elapsed (0.769s cpu)
> IKKhigh <- read.maf(file.choose())
-Reading
-Validating
--Removed 27 duplicated variants
--Non MAF specific values in Variant_Classification column:
  Multi_Hit
  inframe_indel|Missense_Mutation
  3UTR
--Non MAF specific values in Variant_Type column:
  Complex
-Silent variants: 97
-Summarizing
-Processing clinical data
--Missing clinical data
-Finished in 13.2s elapsed (0.465s cpu)
```

```
> oncoplot(maf=IKKlow,top=10)
> oncoplot(maf=IKKhigh,top=10)
> OncogenicPathways(maf= IKKlow)
      Pathway  N n_affected_genes fraction_affected Mutated_samples Fraction_mutated_samples
 1:     Hippo 38                1        0.02631579               9               0.07086614
 2:       MYC 13                1        0.07692308               2               0.01574803
 3:      NRF2  3                1        0.33333333               3               0.02362205
 4:  TGF-Beta  7                2        0.28571429              33               0.25984252
 5:      TP53  6                3        0.50000000              95               0.74803150
 6:       WNT 68                4        0.05882353             110               0.86614173
 7:     NOTCH 71                7        0.09859155              57               0.44881890
 8: Cell_Cycle 15               8        0.53333333              14               0.11023622
 9:      PI3K 29               11        0.37931034              55               0.43307087
10:   RTK-RAS 85               26        0.30588235             111               0.87401575
> OncogenicPathways(maf= IKKhigh)
      Pathway  N n_affected_genes fraction_affected Mutated_samples Fraction_mutated_samples
 1:       MYC 13                1        0.07692308               1               0.03333333
 2:      NRF2  3                1        0.33333333               1               0.03333333
 3:     Hippo 38                2        0.05263158               3               0.10000000
 4:  TGF-Beta  7                2        0.28571429               8               0.26666667
 5:       WNT 68                4        0.05882353              26               0.86666667
 6:      TP53  6                4        0.66666667              25               0.83333333
 7: Cell_Cycle 15               6        0.40000000               7               0.23333333
 8:     NOTCH 71                7        0.09859155              17               0.56666667
 9:      PI3K 29                7        0.24137931              12               0.40000000
10:   RTK-RAS 85               22        0.25882353              24               0.80000000
> IKKlow.vs.IKKhigh <- mafCompare(m1 = IKKlow, m2 = IKKhigh, m1Name = 'Low', m2Name = 'High', minMut = 5)
> print(IKKlow.vs.IKKhigh)
$results
   Hugo_Symbol Low High       pval        or     ci.up     ci.low    adjPval
 1:       ASTE1   8    7 0.01001805 0.2238304 0.8012648 0.06359222 0.4921700
 2:     SLC23A2   6    6 0.01200415 0.2012461 0.8214793 0.04908922 0.4921700
 3:        POLE   7    5 0.05385437 0.2947028 1.2768974 0.07343308 0.7519427
 4:         TTK  12    7 0.05674470 0.3457914 1.1527239 0.11070825 0.7519427
 5:       BRCA2  13    7 0.06792041 0.3775869 1.2442178 0.12308978 0.7519427
 6:         B2M   5    4 0.06844821 0.2694396 1.4533705 0.05383565 0.7519427
 7:      PDGFRA   5    4 0.06844821 0.2694396 1.4533705 0.05383565 0.7519427
 8:        PTEN  14    0 0.07336026       Inf       Inf 0.81936105 0.7519427
 9:     SMARCA4   6    4 0.09872874 0.3253476 1.6812828 0.07119608 0.8723136
10:          AR  30   12 0.10637970 0.4664463 1.1897593 0.18707918 0.8723136
11:       EP300   8    5 0.13154671 0.3391165 1.4311206 0.08882791 0.9133470
12:      TGFBR2  15    7 0.13983790 0.4427883 1.4320047 0.14859127 0.9133470
13:         BLM   9    5 0.14570957 0.3842868 1.5876531 0.10455842 0.9133470
14:      CREBBP  10    5 0.16625170 0.4301706 1.7467397 0.12067300 0.9133470
15:       PBRM1   5    3 0.17910296 0.3718493 2.5384351 0.06752637 0.9133470
16:      ARID1A  21    8 0.20046269 0.5471584 1.6169482 0.19929906 0.9133470
17:       GATA3   9    0 0.20860635       Inf       Inf 0.47031025 0.9133470
18:      DICER1  10    0 0.21047644       Inf       Inf 0.53763774 0.9133470
19:       PTCH1   7    4 0.22327762 0.3821234 1.9125568 0.08914869 0.9133470
20:        KRAS  64   11 0.22358246 1.7484577 4.4203978 0.72223406 0.9133470
21:         ALK  15    6 0.24106449 0.5381326 1.8704627 0.17395952 0.9133470
22:      TMBIM4   8    4 0.24524246 0.4397898 2.1474817 0.10767815 0.9133470
23:       RPL22  17    7 0.25618270 0.5103057 1.6264330 0.17504174 0.9133470
24:       ERBB3   9    4 0.27465524 0.4983789 2.3859382 0.12660822 0.9384054
25:         RET   7    0 0.34778915       Inf       Inf 0.33941406 1.0000000
26:        ATRX   6    3 0.37458847 0.4490883 2.9473353 0.08898161 1.0000000
27:        CIITA   6    3 0.37458847 0.4490883 2.9473353 0.08898161 1.0000000
28:       ASXL1  17    6 0.39089864 0.6202814 2.1261121 0.20487592 1.0000000
29:       FBXW7  17    6 0.39089864 0.6202814 2.1261121 0.20487592 1.0000000
30:      MAP3K1   7    3 0.40433587 0.5275093 3.3623781 0.11122247 1.0000000
31:       SETD2   7    3 0.40433587 0.5275093 3.3623781 0.11122247 1.0000000
```

```
32:        SMO   7    3 0.40433587 0.5275093   3.3623781 0.11122247 1.0000000
33:       MTOR   8    3 0.44087494 0.6072447   3.7844407 0.13407620 1.0000000
34:      TAF1B   8    3 0.44087494 0.6072447   3.7844407 0.13407620 1.0000000
35:      PALB2  11    1 0.46455162 2.7368742 122.3349771 0.36954367 1.0000000
36:      ERBB4  12    4 0.51041225 0.6801031   3.1255163 0.18595751 1.0000000
37:     NOTCH2  12    4 0.51041225 0.6801031   3.1255163 0.18595751 1.0000000
38:     NOTCH4  15    5 0.54214769 0.6715161   2.5836987 0.20622828 1.0000000
39:       RAF1   5    0 0.58406150       Inf         Inf 0.21393275 1.0000000
40:      RNF43  20    6 0.58862733 0.7491340   2.5274659 0.25342227 1.0000000
41:    CCDC150   5    2 0.61928362 0.5761261   6.3445321 0.08857889 1.0000000
42:      CHEK2   5    2 0.61928362 0.5761261   6.3445321 0.08857889 1.0000000
43:      ERBB2   5    2 0.61928362 0.5761261   6.3445321 0.08857889 1.0000000
44:      FGFR4   5    2 0.61928362 0.5761261   6.3445321 0.08857889 1.0000000
45:       MEN1   5    2 0.61928362 0.5761261   6.3445321 0.08857889 1.0000000
46:      RUNX1   6    2 0.64901575 0.6959969   7.4060780 0.11627587 1.0000000
47:       TP53  77   20 0.67685460 0.7712589   1.8986671 0.29639510 1.0000000
48:       IDH1   7    2 0.68199414 0.8177800   8.4846651 0.14482613 1.0000000
49:      CDK12   9    1 0.68844020 2.2028937 100.2063382 0.28480076 1.0000000
50:      SF3B1   9    1 0.68844020 2.2028937 100.2063382 0.28480076 1.0000000
51:       GNAS   9    3 0.70102725 0.6882524   4.2132295 0.15745888 1.0000000
52:        NF1   9    3 0.70102725 0.6882524   4.2132295 0.15745888 1.0000000
53:       AIM2  10    3 0.71489808 0.7706056   4.6490741 0.18137706 1.0000000
54:      BRCA1  10    3 0.71489808 0.7706056   4.6490741 0.18137706 1.0000000
55:      AMER1  11    3 0.73205699 0.8543215   5.0923870 0.20574190 1.0000000
56:      RAD50  11    3 0.73205699 0.8543215   5.0923870 0.20574190 1.0000000
57:       BRAF  21    4 0.78724197 1.2857464   5.5934097 0.38553581 1.0000000
58:     PIK3CA  26    5 0.80052555 1.2851146   4.7152825 0.42508788 1.0000000
59:        APC  88   21 1.00000000 0.9672364   2.4501684 0.35636385 1.0000000
60:     CTNNB1  10    2 1.00000000 1.1952751  11.8250345 0.23477173 1.0000000
61:     NOTCH3  15    3 1.00000000 1.2039815   6.9451405 0.30803454 1.0000000
62:       NRAS  10    2 1.00000000 1.1952751  11.8250345 0.23477173 1.0000000
63:     ARID1B   8    2 1.00000000 0.9415386   9.5797972 0.17414891 1.0000000
64:      ARID2   9    2 1.00000000 1.0673368  10.6929960 0.20416172 1.0000000
65:        ATM  21    5 1.00000000 0.9906258   3.6914454 0.32001574 1.0000000
66:        ATR  17    4 1.00000000 1.0045168   4.4470226 0.29273495 1.0000000
67:     COBLL1   8    1 1.00000000 1.9427024  89.4194012 0.24365629 1.0000000
68:       CTCF   7    1 1.00000000 1.6867822  78.8112904 0.20337251 1.0000000
69:      GNA11   5    1 1.00000000 1.1872805  58.1277446 0.12575888 1.0000000
70:       IDH2   5    1 1.00000000 1.1872805  58.1277446 0.12575888 1.0000000
71:       JAK1   5    1 1.00000000 1.1872805  58.1277446 0.12575888 1.0000000
72:      KMT2A  12    2 1.00000000 1.4576530  14.1494673 0.29780844 1.0000000
73:       MSH2  19    4 1.00000000 1.1425671   5.0096955 0.33828153 1.0000000
74:       MSH6  14    3 1.00000000 1.1142903   6.4694426 0.28173609 1.0000000
75:        NBN   5    1 1.00000000 1.1872805  58.1277446 0.12575888 1.0000000
76:     NOTCH1  18    4 1.00000000 1.0729175   4.7257866 0.31529577 1.0000000
77:     PIK3R1   8    1 1.00000000 1.9427024  89.4194012 0.24365629 1.0000000
78:       POLQ  11    2 1.00000000 1.3253537  12.9772713 0.26602150 1.0000000
79:       ROS1   8    2 1.00000000 0.9415386   9.5797972 0.17414891 1.0000000
80:      SMAD4  18    4 1.00000000 1.0729175   4.7257866 0.31529577 1.0000000
81:      TEAD2   9    2 1.00000000 1.0673368  10.6929960 0.20416172 1.0000000
82:       TSC2   8    2 1.00000000 0.9415386   9.5797972 0.17414891 1.0000000
    Hugo_Symbol Low High       pval        or      ci.up     ci.low   adjPval

$SampleSummary
   Cohort SampleSize
1:    Low        127
2:   High         30

> forestPlot(mafCompareRes = IKKlow.vs.IKKhigh, pVal = 0.05, color = c('royalblue', 'maroon'), geneFontSize = 0.8)
> genes = c("ASTE1", "SLC23A2", "POLE", "TTK", "BRCA2", "B2M", "PDGFRA", "PTEN", "SMARCA4", "AR")
> coBarplot(m1 = IKKlow, m2 = IKKhigh, m1Name = 'Low', m2Name = 'High', genes = genes)
```

```
> IKKall <- read.maf(file.choose())
-Reading
-Validating
--Removed 199 duplicated variants
--Non MAF specific values in Variant_Classification column:
  Multi_Hit
  3UTR
  inframe_indel|stop_gained
  inframe_indel
  inframe_indel|Missense_Mutation

--Non MAF specific values in Variant_Type column:
  Complex
-Silent variants: 613
-Summarizing
-Processing clinical data
--Missing clinical data
-Finished in 3.460s elapsed (0.270s cpu)
> lollipopPlot(maf = IKKall, gene = 'AR',  AACol = "VAF")
2 transcripts available. Use arguments refSeqID or proteinID to manually specify tx name.
   HGNC    refseq.ID   protein.ID aa.length
1:   AR    NM_000044    NP_000035       920
2:   AR NM_001011645 NP_001011645       388
Using longer transcript NM_000044 for now.

> IKKpunctatelow <- read.maf(file.choose())
-Reading
-Validating
Error in validateMaf(maf = maf, isTCGA = isTCGA, rdup = removeDuplicatedVariants,  :
  missing required fields from MAF: Hugo_Symbol
> IKKpunctatelow <- read.maf(file.choose())
-Reading
-Validating
--Removed 137 duplicated variants
--Non MAF specific values in Variant_Classification column:
  Multi_Hit
  3UTR
  inframe_indel|stop_gained
  inframe_indel
  inframe_indel|Missense_Mutation
--Non MAF specific values in Variant_Type column:
  Complex
-Silent variants: 425
-Summarizing
-Processing clinical data
--Missing clinical data
-Finished in 22.0s elapsed (0.854s cpu)
> IKKpunctatehigh <- read.maf(file.choose())
-Reading
-Validating
--Removed 5 duplicated variants
--Non MAF specific values in Variant_Classification column:
  Multi_Hit
--Non MAF specific values in Variant_Type column:
  Complex
-Silent variants: 12
-Summarizing
-Processing clinical data
--Missing clinical data
-Finished in 6.338s elapsed (0.352s cpu)
> oncoplot(maf=IKKpunctatelow,top=10)
```

```
> oncoplot(maf=IKKpunctatehigh,top=10)
> OncogenicPathways(maf= IKKpunctatelow)
     Pathway  N n_affected_genes fraction_affected Mutated_samples Fraction_mutated_samples
 1:      NRF2  3              1        0.33333333               3               0.02040816
 2:     Hippo 38              2        0.05263158              12               0.08163265
 3:       MYC 13              2        0.15384615               3               0.02040816
 4:  TGF-Beta  7              2        0.28571429              38               0.25850340
 5:       WNT 68              4        0.05882353             129               0.87755102
 6:      TP53  6              4        0.66666667             112               0.76190476
 7:     NOTCH 71              7        0.09859155              72               0.48979592
 8: Cell_Cycle 15             8        0.53333333              20               0.13605442
 9:      PI3K 29             11        0.37931034              63               0.42857143
10:   RTK-RAS 85             28        0.32941176             124               0.84353741
> OncogenicPathways(maf= IKKpunctatehigh)
     Pathway  N n_affected_genes fraction_affected Mutated_samples Fraction_mutated_samples
1: Cell_Cycle 15              1        0.06666667               1               0.09090909
2:      NRF2  3              1        0.33333333               1               0.09090909
3:       WNT 68              2        0.02941176               8               0.72727273
4:  TGF-Beta  7              2        0.28571429               3               0.27272727
5:     NOTCH 71              3        0.04225352               3               0.27272727
6:      TP53  6              3        0.50000000               9               0.81818182
7:      PI3K 29              4        0.13793103               5               0.45454545
8:   RTK-RAS 85             11        0.12941176              11               1.00000000
> IKKpunctatelow.vs.IKKpunctatehigh <- mafCompare(m1 = IKKpunctatelow, m2 = IKKpunctatehigh, m1Name = 'Low', m2Name = 'High', minMut = 5)
> print(IKKpunctatelow.vs.IKKpunctatehigh)
$results
   Hugo_Symbol Low High       pval        or      ci.up     ci.low adjPval
 1:     PDGFRA   6    3 0.01693304 0.1168469  0.8512627 0.01983251       1
 2:       KRAS  66    9 0.02602932 0.1828151  0.9264554 0.01861796       1
 3:        RET   5    2 0.07655128 0.1621296  1.9238916 0.02227786       1
 4:     PIK3R1   7    2 0.12135753 0.2286777  2.5660751 0.03550370       1
 5:       ROS1   8    2 0.14588678 0.2625954  2.8938770 0.04231794       1
 6:      SMAD4  19    3 0.18333364 0.3988933  2.5354896 0.08560004       1
 7:       TP53  89    9 0.20833360 0.3429801  1.7419962 0.03485732       1
 8:      RNF43  26    0 0.21329377       Inf        Inf 0.50028993       1
 9:       BRAF  25    0 0.21481915       Inf        Inf 0.47609327       1
10:      AMER1  12    2 0.25246992 0.4031851  4.2530257 0.07060773       1
11:     NOTCH3  19    0 0.36280436       Inf        Inf 0.33875949       1
12:      CHEK2   6    1 0.40272353 0.4287501 21.5081086 0.04447555       1
13:       MEN1   6    1 0.40272353 0.4287501 21.5081086 0.04447555       1
14:       CTCF   7    1 0.44623374 0.5028682 24.7613140 0.05468634       1
15:      PBRM1   7    1 0.44623374 0.5028682 24.7613140 0.05468634       1
16:     PIK3CA  28    3 0.45224205 0.6295333  3.9164291 0.13934373       1
17:      GATA3   8    1 0.48684326 0.5780276 28.0613262 0.06507063       1
18:       IDH1   8    1 0.48684326 0.5780276 28.0613262 0.06507063       1
19:     DICER1   9    1 0.52472732 0.6542535 31.4091629 0.07561369       1
20:      ARID2  10    1 0.56005164 0.7315656 34.8058715 0.08632258       1
21:      PTCH1  10    1 0.56005164 0.7315656 34.8058715 0.08632258       1
22:      TAF1B  10    1 0.56005164 0.7315656 34.8058715 0.08632258       1
23:        NF1  11    1 0.59297295 0.8099986 38.2525304 0.09719398       1
24:       NRAS  11    1 0.59297295 0.8099986 38.2525304 0.09719398       1
25:       POLE  11    1 0.59297295 0.8099986 38.2525304 0.09719398       1
26:    SLC23A2  11    1 0.59297295 0.8099986 38.2525304 0.09719398       1
27:     TMBIM4  11    1 0.59297295 0.8099986 38.2525304 0.09719398       1
28:        BLM  14    0 0.60027905       Inf        Inf 0.23362621       1
29:      KMT2A  14    0 0.60027905       Inf        Inf 0.23362621       1
30:       AIM2  13    0 0.60133652       Inf        Inf 0.21353998       1
31:      EP300  13    0 0.60133652       Inf        Inf 0.21353998       1
32:       POLQ  13    0 0.60133652       Inf        Inf 0.21353998       1
33:     CREBBP  15    0 0.60146870       Inf        Inf 0.25402248       1
34:      ERBB4  16    0 0.60462722       Inf        Inf 0.27472624       1
```

```
35:     NOTCH2  16    0 0.60462722       Inf        Inf 0.27472624        1
36:       MSH6  17    0 0.60949977       Inf        Inf 0.29574428        1
37:      FBXW7  21    2 0.66303229 0.7514911  7.6238349 0.14073376        1
38:       MSH2  21    2 0.66303229 0.7514911  7.6238349 0.14073376        1
39:        ATM  25    1 0.69324948 2.0416638 92.4064544 0.26796002        1
40:         AR  40    2 0.72867399 1.6773149 16.6166388 0.32669716        1
41:        ALK  20    1 1.00000000 1.5708943 71.6962555 0.20272038        1
42:     ARID1B  10    0 1.00000000       Inf        Inf 0.15502690        1
43:        ATR  20    1 1.00000000 1.5708943 71.6962555 0.20272038        1
44:      CDK12  10    0 1.00000000       Inf        Inf 0.15502690        1
45:     MAP3K1  10    0 1.00000000       Inf        Inf 0.15502690        1
46:       PTEN  13    1 1.00000000 0.9703327 45.3001732 0.11942897        1
47:      RAD50  13    1 1.00000000 0.9703327 45.3001732 0.11942897        1
48:      SETD2  10    0 1.00000000       Inf        Inf 0.15502690        1
49:      SF3B1  10    0 1.00000000       Inf        Inf 0.15502690        1
50:    SMARCA4  10    0 1.00000000       Inf        Inf 0.15502690        1
51:        SMO  10    0 1.00000000       Inf        Inf 0.15502690        1
52:       TSC2  10    0 1.00000000       Inf        Inf 0.15502690        1
53:        TTK  18    1 1.00000000 1.3927159 63.8606786 0.17801415        1
54:        APC 102    8 1.00000000 0.8508612  3.7578728 0.13899153        1
55:     ARID1A  27    2 1.00000000 1.0124222 10.1566173 0.19323340        1
56:      ASTE1  14    1 1.00000000 1.0523028 48.9034785 0.13078601        1
57:      ASXL1  22    1 1.00000000 1.7547234 79.7819907 0.22819906        1
58:       ATRX   9    0 1.00000000       Inf        Inf 0.13609689        1
59:        B2M   9    0 1.00000000       Inf        Inf 0.13609689        1
60:      BRCA1  12    1 1.00000000 0.8896011 41.7502498 0.10822889        1
61:      BRCA2  19    1 1.00000000 1.4811152 67.7479275 0.19027863        1
62:      CASP5   5    0 1.00000000       Inf        Inf 0.06328963        1
63:    CCDC150   7    0 1.00000000       Inf        Inf 0.09909482        1
64:      CIITA   9    0 1.00000000       Inf        Inf 0.13609689        1
65:     COBLL1   9    0 1.00000000       Inf        Inf 0.13609689        1
66:     CTNNB1  12    0 1.00000000       Inf        Inf 0.19374529        1
67:     DNMT3A   6    0 1.00000000       Inf        Inf 0.08103391        1
68:      ERBB2   7    0 1.00000000       Inf        Inf 0.09909482        1
69:      ERBB3  12    1 1.00000000 0.8896011 41.7502498 0.10822889        1
70:      FGFR1   6    0 1.00000000       Inf        Inf 0.08103391        1
71:      FGFR2   7    0 1.00000000       Inf        Inf 0.09909482        1
72:      FGFR3   6    0 1.00000000       Inf        Inf 0.08103391        1
73:      FGFR4   7    0 1.00000000       Inf        Inf 0.09909482        1
74:      GNA11   6    0 1.00000000       Inf        Inf 0.08103391        1
75:       GNAS  12    0 1.00000000       Inf        Inf 0.19374529        1
76:        HGF   5    0 1.00000000       Inf        Inf 0.06328963        1
77:       IDH2   6    0 1.00000000       Inf        Inf 0.08103391        1
78:       JAK1   6    0 1.00000000       Inf        Inf 0.08103391        1
79:     MAP2K4   6    0 1.00000000       Inf        Inf 0.08103391        1
80:       MLH1   5    0 1.00000000       Inf        Inf 0.06328963        1
81:       MTOR  11    0 1.00000000       Inf        Inf 0.17424224        1
82:        NBN   6    0 1.00000000       Inf        Inf 0.08103391        1
83:     NOTCH1  21    1 1.00000000 1.6620859 75.7071114 0.21535957        1
84:     NOTCH4  19    1 1.00000000 1.4811152 67.7479275 0.19027863        1
85:      NTRK1   6    0 1.00000000       Inf        Inf 0.08103391        1
86:      PALB2  12    0 1.00000000       Inf        Inf 0.19374529        1
87:     PIK3CB   5    0 1.00000000       Inf        Inf 0.06328963        1
88:        RB1   5    0 1.00000000       Inf        Inf 0.06328963        1
89:      RPL22  23    1 1.00000000 1.8488417 83.9224367 0.24124265        1
90:      RUNX1   8    0 1.00000000       Inf        Inf 0.11745199        1
91:      TEAD2  11    0 1.00000000       Inf        Inf 0.17424224        1
92:     TGFBR2  21    1 1.00000000 1.6620859 75.7071114 0.21535957        1
    Hugo_Symbol Low High       pval        or      ci.up     ci.low adjPval

$SampleSummary
```

```
   Cohort SampleSize
1:    Low        147
2:   High         11

> forestPlot(mafCompareRes = IKKpunctatelow.vs.IKKpunctatehigh, pVal = 0.05, color = c('royalblue', 'maroon'), geneFontSize = 0.8)
> genes = c("PDGFRA", "KRAS", "RET", "PIK3R1", "ROS1", "SMAD4", "TP53", "RNF43", "BRAF", "AMER1")
> coBarplot(m1 = IKKpunctatelow, m2 = IKKpunctatehigh, m1Name = 'Low', m2Name = 'High', genes = genes)
> IKKall <- read.maf(file.choose())
-Reading
-Validating
--Removed 199 duplicated variants
--Non MAF specific values in Variant_Classification column:
  Multi_Hit
  3UTR
  inframe_indel|stop_gained
  inframe_indel
  inframe_indel|Missense_Mutation

--Non MAF specific values in Variant_Type column:
  Complex
-Silent variants: 613
-Summarizing
-Processing clinical data
--Missing clinical data
-Finished in 14.9s elapsed (0.735s cpu)
> plotmafSummary(maf = IKKall, rmOutlier = TRUE, addStat = 'median', dashboard = TRUE, titvRaw = FALSE)
> plotVaf(maf = IKKall, vafCol = 'VAF', top = 10)
```

Supplementary Table 4. Univariate and multivariate analysis in patients undergoing surgery for colorectal cancer

Clinicopathological characteristics	Univariate HR (95% CI)	*P*	Multivariate analysis	*p*
**Age (<65/>65)**	1.25 (0.80 – 1.31)	0.859		
**Sex (Female/Male)**	1.12 (0.89 – 1.42)	0.348		
**Type of surgery (Elective/Emergency)**	1.74 (1.33 – 2.28)	<0.001	1.69 (1.23 – 2.33)	**0.002**
**Tumour location (Right/Left/Rectum)**	0.74 (0.88 – 1.20)	0.725		
**Tumour Characteristics**				
**TNM stage (I/II/III)**	2.63 (2.15 – 3.23)	<0.001	2.42 (1.90 – 3.08)	**<0.001**
**Tumour differentiation (Mod-well/poor)**	1.69 (1.22 – 2.35)	<0.001		
**Venous invasion (No/Yes)**	1.67 (1.31 – 2.13)	<0.001	1.51 (1.12 – 2.02)	**0.007**
**Margin involvement (No/Yes)**	3.73 (1.71 – 8.14)	<0.001	2.26 (1.43 – 3.57)	**0.001**
**Necrosis (Absent/Present)**	1.13 (0.67 – 1.91)	0.088		
**Proliferation (High/Low)**	0.79 (0.48 – 1.29)	<0.001		
**MMR status (Proficient/Deficient)**	1.10 (0.55 – 2.16)	0.176		
**Tumour microenvironment**				
**Klintrup-Mäkinen grade (Weak/Strong)**	0.51 (0.28 – 0.93)	<0.001	0.47 (0.32-0.68)	**<0.001**
**Tumour stroma percentage (Low/High)**	2.01 (1.22 – 3.54)	<0.001	1.58 (1.16-2.14)	**0.005**

**Adjuvant therapy (No/Yes)**	1.05 (0.63 – 1.74)	0.994		
**Cytoplasmic IKK-α (Low/High)**	1.56 (0.87 – 2.78)	0.240		
**Punctate IKK-α (Low/High)**	2.22 (1.14 – 4.30)	<0.001	1.97 (1.26 –3.08)	**0.006**

Supplementary Figure 1. Altered CHUK gene signature and survival in patients from the TCGA panCancer atlas colorectal cohort

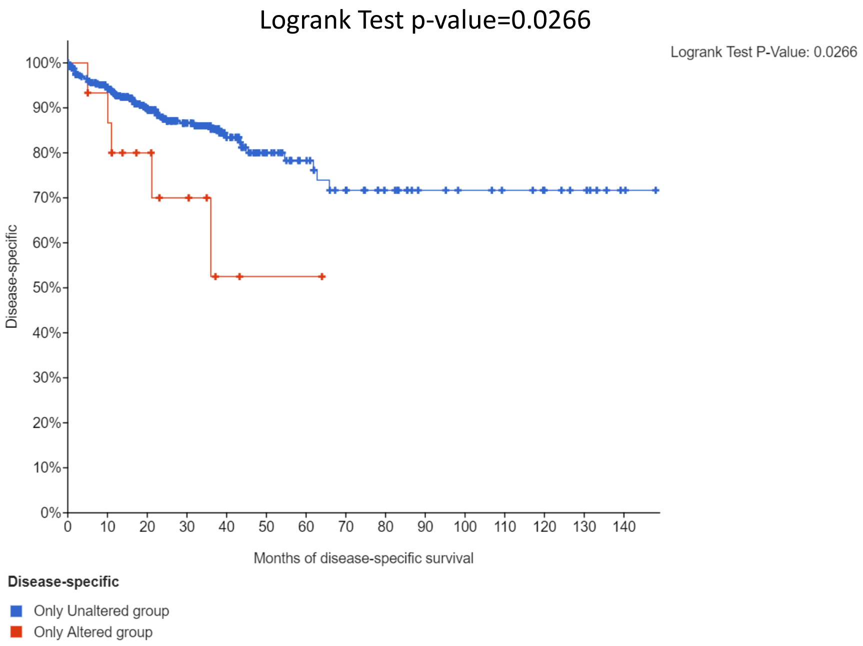

Supplementary Figure 2. Mutational cohort characteristics

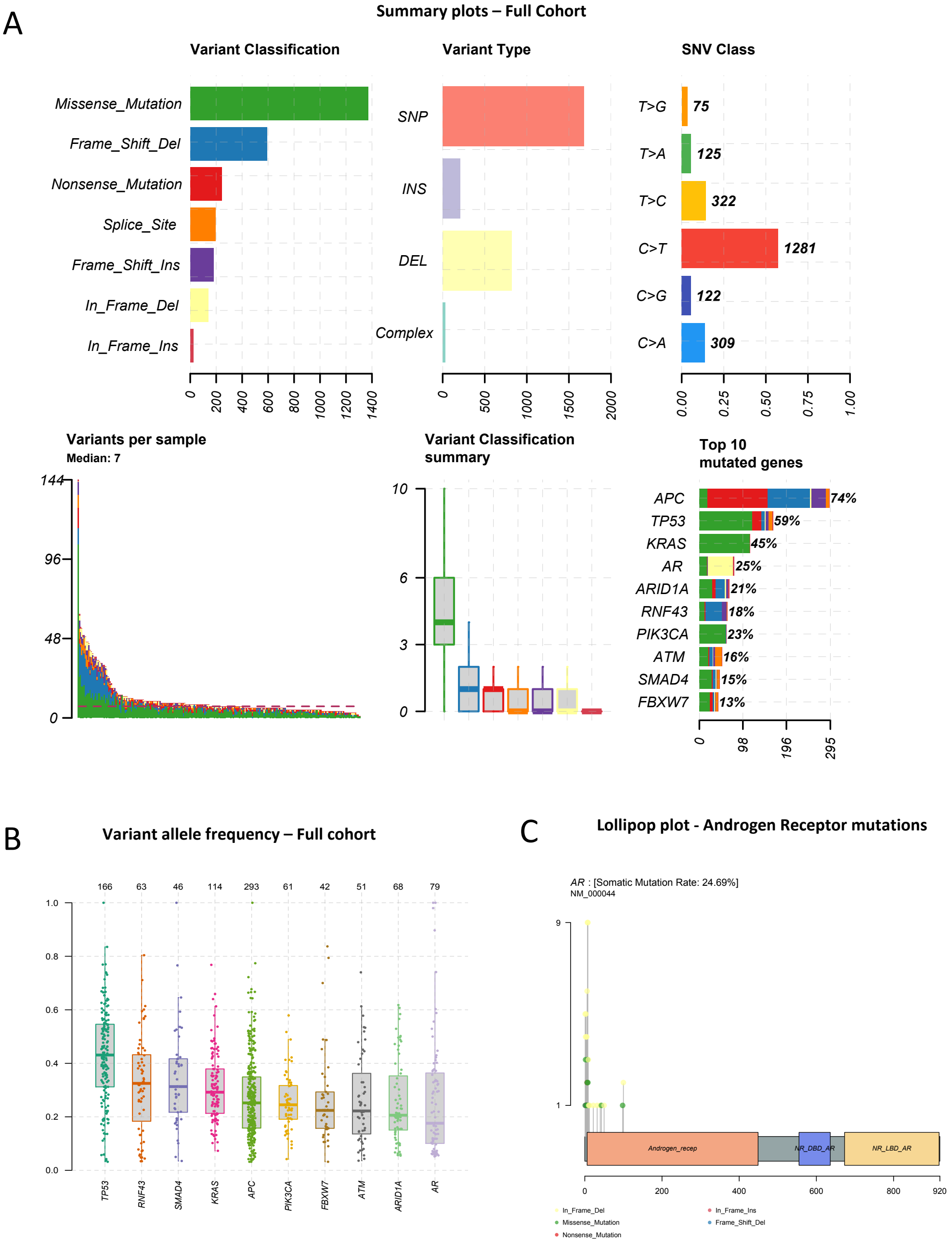

Supplementary Figure 3. Mutations in oncogenic pathways in low vs high cytoplasmic and low vs. high punctate IKKα

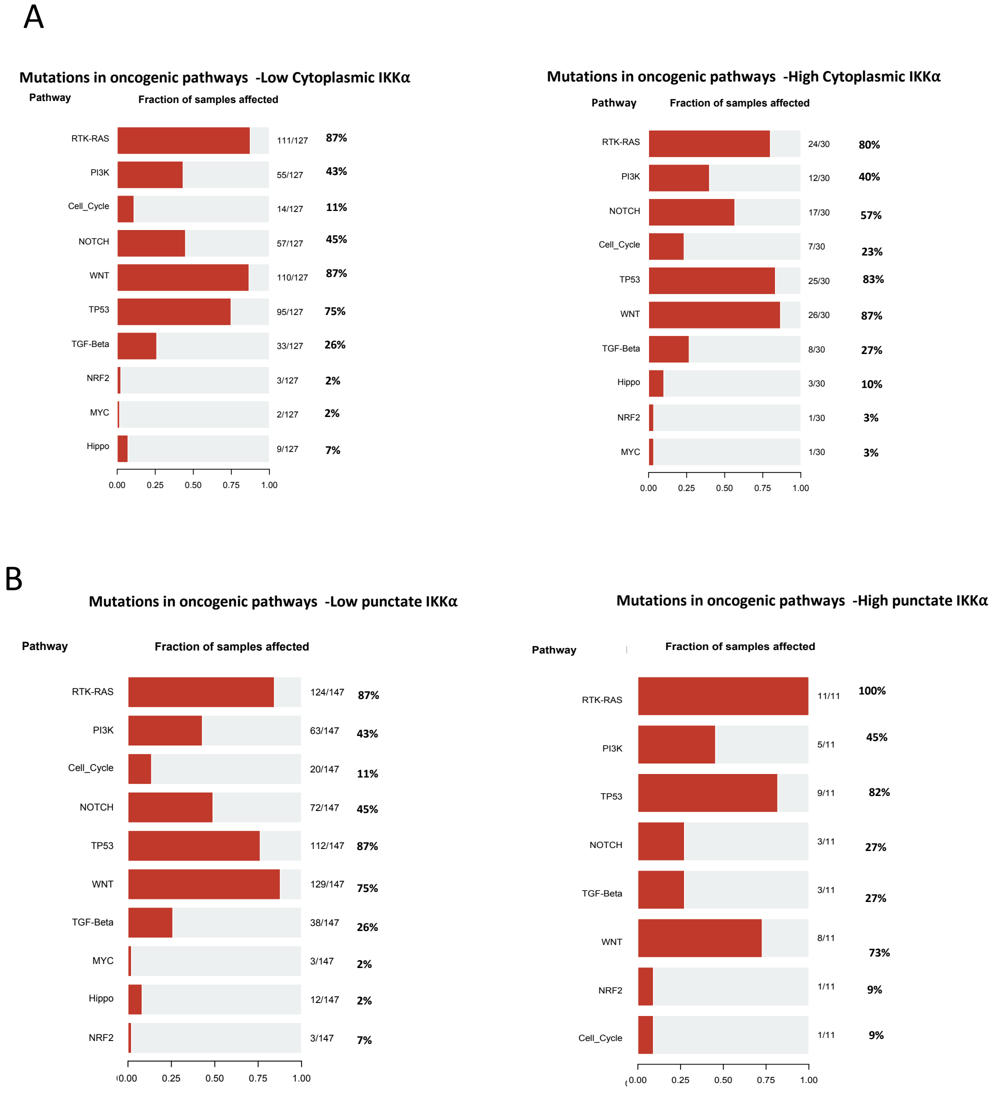

Supplement: Supplementary file 2 — Supplementary Tables and Figures [file 41416_2022_1729_MOESM2_ESM.pdf]
